# Hereditary Angioedema with Normal C1 Inhibitor: an Updated International Consensus Paper on Diagnosis, Pathophysiology, and Treatment

**DOI:** 10.1007/s12016-025-09027-4

**Published:** 2025-03-07

**Authors:** Bruce L. Zuraw, Konrad Bork, Laurence Bouillet, Sandra C. Christiansen, Henriette Farkas, Anastasios E. Germenis, Anete S. Grumach, Allen Kaplan, Alberto López-Lera, Markus Magerl, Marc A. Riedl, Adil Adatia, Aleena Banerji, Stephen Betschel, Isabelle Boccon-Gibod, Maria Bova, Henrik Balle Boysen, Teresa Caballero, Mauro Cancian, Anthony J. Castaldo, Danny M. Cohn, Deborah Corcoran, Christian Drouet, Atsushi Fukunaga, Michihiro Hide, Constance H. Katelaris, Philip H. Li, Hilary Longhurst, Jonny Peter, Fotis Psarros, Avner Reshef, Bruce Ritchie, Christine N. Selva, Andrea Zanichelli, Marcus Maurer

**Affiliations:** 1 Department of Medicine, Division of Allergy & Immunology, University of California San Diego, 9500 Gilman Drive, Mail Code 0732, La Jolla, CA 92093 USA; 2 Medicine Service, San Diego VA Healthcare, San Diego, USA; 3 Department of Dermatology, University Medical Center, Johannes Gutenberg University Mainz, Mainz, Germany; 4 University Grenoble Alpes, T-RAIG Unit, CNRS, UMR 5525, TIMC, Grenoble, France; 5 French National Reference Center for Angioedema (CREAK), Internal Medicine Department, Grenoble University Hospital, Grenoble, France; 6 Hungarian Angioedema Center of Reference and Excellence, Department of Internal Medicine and Haematology, Semmelweis University, Budapest, Hungary; 7 Department of Immunology & Histocompatibility, School of Medicine, University of Thessaly, Larissa, Greece; 8 Angioedema Center of Reference and Excellence (ACARE), Centro Universitario Faculdade de Medicina ABC (CEUFMABC), São Paulo, Brazil; 9 Medical University of South Carolina, Charleston, SC USA; 10 Hospital La Paz Institute for Health Research (IdiPAZ), CIBERER (U754), Madrid, Spain; 11 Angioedema Center of Reference and Excellence (ACARE), Institute of Allergology, Charité – Universitätsmedizin Berlin, corporate member of Freie Universität Berlin and Humboldt-Universität Zu Berlin, Berlin, Germany; 12 Fraunhofer Institute for Translational Medicine and Pharmacology ITMP, Immunology and Allergology, Berlin, Germany; 13 Division of Pulmonary Medicine, Department of Medicine, University of Alberta, Edmonton, AB Canada; 14 Department of Medicine, Division of Rheumatology, Allergy and Immunology, Massachusetts General Hospital, Boston, MA USA; 15 Division of Clinical Immunology and Allergy, University of Toronto, Toronto, ON Canada; 16 Division of Internal Medicine 2, Department of Medicine and Medical Specialties, A. Cardarelli Hospital, Naples, Italy; 17 HAE International (HAEi), Fairfax, VA USA; 18 US Hereditary Angioedema Association (HAEA), Fairfax, VA USA; 19 Department of Allergy, La Paz University Hospital, Madrid, Spain; 20 Department of Systems Medicine, University of Padua, Padua, Italy; 21 Department of Vascular Medicine, Amsterdam Cardiovascular Sciences, Amsterdam UMC, University of Amsterdam, Amsterdam, the Netherlands; 22 Institut Cochin, Université Paris Cité, INSERM U1016, Paris, France; 23 Department of Dermatology, Division of Medicine for Function and Morphology of Sensory Organs, Faculty of Medicine, Osaka Medical and Pharmaceutical University, Takatsuki-City, Osaka, Japan; 24 Department of Dermatology, Hiroshima City Hiroshima Citizens Hospital, Hiroshima, Japan; 25 Department of Dermatology, Hiroshima University, Hiroshima, Japan; 26 Immunology & Allergy Unit, Dept of Medicine, Campbelltown Hospital and Western Sydney University, Sydney, Australia; 27 Division of Rheumatology and Clinical Immunology, Department of Medicine, Queen Mary Hospital, The University of Hong Kong, Hong Kong, Hong Kong; 28 Department of Medicine, University of Auckland and Department of Immunology, Auckland City Hospital, Auckland, New Zealand; 29 Division of Allergy and Clinical Immunology, Department of Medicine, Groote Schuur Hospital, University of Cape Town, Cape Town, South Africa; 30 Allergy and Immunology Unit, University of Cape Town Lung Institute, Cape Town, South Africa; 31 Department of Allergy, Athens Naval Hospital, Athens, Greece; 32 Angioedema Research Unit, Barzilai University Medical Center, Ashkelon, Israel; 33 Division of Hematology, Department of Medicine, University of Alberta, Edmonton, Alberta, Canada; 34 Operative Unit of Medicine, Angioedema Center, IRCCS Policlinico San Donato, San Donato Milanese, Milan, Italy; 35 Dipartimento Di Scienze Biomediche Per La Salute, University of Milan, Milan, Italy

**Keywords:** HAE, HAE-C1INH, HAE-nC1INH, Bradykinin, Diagnosis, Pathophysiology, Treatment

## Abstract

Hereditary angioedema (HAE) has been recognized for almost 150 years. The newest form of HAE, where C1 inhibitor levels are normal (HAE-nC1INH), was first described in 2000. Over the last two decades, new types of apparent non-mast cell–mediated angioedema with normal quantity and activity of C1INH have been described, in some cases with proven genetic pathogenic variants that co-segregate with angioedema expression within families. Like HAE due to C1INH deficiency, HAE-nC1INH patients are at risk of serious morbidity and mortality. Therefore, proactive management and treatment of HAE-nC1INH patients after an expert physician diagnosis is critically important. The underlying pathophysiology responsible for the angioedema has also been clarified in some of the HAE-nC1INH types. While several clinical guidelines and practice parameters including HAE-nC1INH have been published, we have made substantial progress in our understanding encompassing diagnostic criteria, pathophysiology, and treatment outcomes. HAE International (HAEi) and the US HAE Association (HAEA) convened a symposium of global HAE-nC1INH experts to synthesize our current knowledge in the area. Given the paucity of high-level evidence in HAE-nC1INH, all recommendations are based on expert opinion. This review and expert opinion on the best practice approach to diagnosing and treating HAE-nC1INH will support physicians to better manage patients with HAE-nC1INH.

## Introduction

Angioedema is defined as intermittent, localized, and self-limited swelling of the subcutaneous and/or submucosal tissue. Multiple sites may be involved. It may occur in isolation, accompanied by hives (also known as wheals) or as part of a systemic allergic reaction. Angioedema may occur at any age developing spontaneously or in response to a triggering factor. Symptoms may persist for a few hours or may last days, resolving spontaneously or after treatment. All types of angioedema present with relatively similar signs and symptoms although there are differences between the various forms.

The proximate cause of all angioedema is attributed to increased vascular endothelial permeability [1, 2]. The underlying mechanisms, while heterogeneous and complex, ultimately result in enhanced vascular permeability leading to tissue swelling. Angioedema may result from mediators associated with mast cell activity; excess production of bradykinin due to either activation of the kallikrein-kinin system (KKS) or direct cleavage of kininogens by non- contact system proteases; reduced catabolism of bradykinin, as in some types of drug-induced angioedema; or intrinsic dysfunction of the vascular endothelium [3, 4].

Hereditary angioedema (HAE) has been recognized for almost 150 years [5]. In 1963, Donaldson reported that HAE patients were characterized by decreased plasma levels of C1INH [6], a type of HAE now known as HAE-C1INH. Over the last two decades, new types of apparent non-mast cell mediated angioedema with normal quantity and activity of C1INH were described, in some cases with proven genetic pathogenic variants co-segregating with family and individual angioedema expression [4, 7]. While several clinical guidelines and practice parameters regarding this new type of HAE have been published [8–13], substantial progress has been made encompassing diagnostic criteria, pathophysiology, and treatment outcomes.

In the summer of 2023, HAE International (HAEi) and the US HAE Association (HAEA) enlisted 31 HAE with normal C1INH (HAE-nC1INH) global experts to synthesize our current knowledge in the area in order to present a best practice approach to the diagnosis and management of HAE-nC1INH. Preliminary drafts of the findings were presented and discussed in a symposium held on September 1, 2023 in Munich, Germany. The current manuscript reflects four rounds of revisions of these original drafts. Given the paucity of high-level evidence, all recommendations in this paper are based on expert opinion.

## Section 1: Classification and Diagnosis of HAE-nC1INH

### Classification of Recurrent Angioedema Without Hives

Recurrent angioedema without hives encompasses a range of diagnoses with markedly different prognoses and treatments. Several different classification schemes of angioedema have recently been proposed [14, 15]. When evaluating a patient with angioedema, two of the most important criteria to consider are (1) whether the mechanism of swelling is from mast cell activation or another cause (especially bradykinin but potentially including other mast cell–independent mediators or mechanisms affecting vascular permeability) and (2) whether it is hereditary or acquired.

Mast cell–mediated angioedema can result from type I hypersensitivity reactions (antigen bound to specific IgE on mast cells), direct mast cell activation, or as part of chronic urticaria [16]. While chronic urticaria most often includes hives, a significant minority (~ 10%) of these patients present only with angioedema [17]. Mast cell–mediated angioedema without hives may be a separate disorder than chronic urticaria.

A number of drugs can cause angioedema through a variety of mechanisms in the absence of specific IgE. The drugs most commonly associated with angioedema include angiotensin converting enzyme (ACE) inhibitors, other kininase (such as neprilysin or dipeptidyl peptidase IV [DPPIV]) inhibitors, tissue plasminogen activators, and non-steroidal anti-inflammatory drugs (NSAIDs) [18–21].

HAE includes a group of rare genetically transmitted disorders characterized by recurrent angioedema without hives. It should be recognized that hives are common and can occur coincidentally in patients with a separate diagnosis of recurrent angioedema. Biochemical abnormalities in HAE were first identified in 1962 and 1963 [6, 22] and subsequently shown to be autosomal dominant with high penetrance. HAE-C1INH is caused by pathogenic variants in the gene encoding C1INH (*SERPING1*) resulting in reduced functional levels of C1INH and leading to dysregulated production of bradykinin [23–26].

In 2000, another type of HAE with autosomal dominant inheritance, normal C1INH function, and variable penetrance was identified [27, 28]. While originally designated HAE type III, the name was later changed to HAE-nC1INH [8]. At the time of the Symposium, mutations in six different genes had been linked to HAE-nC1INH, although many patients still lack an identified pathogenic variant [29–34] (Table 1). The genes associated with HAE-nC1INH include *F12*, the gene for coagulation factor XII (FXII); *PLG*, the gene for plasminogen; *ANGPT1*, the gene for angiopoietin-1; *KNG1*, the gene for kininogen-1; *MYOF*, the gene for myoferlin; and *HS3ST6*, the gene for heparan sulfate glucosamine 3-*O*-sulfotransferase-6 (HS3OST6). In the past year, pathogenic variants in two additional genes have been linked to HAE-nC1INH in families that also experienced hives, specifically *CPN1*, the gene for carboxypeptidase N (CPN), and *DAB2IP*, the gene for disabled homolog 2 interacting protein (DAB2IP) [35, 36]. When the underlying pathogenic variant is known, HAE-nC1INH is named after the relevant mutated protein (i.e., HAE-nC1INH caused by a plasminogen pathogenic variant is called HAE-PLG). HAE-FXII and likely HAE-PLG have been clearly shown to be bradykinin-mediated [37–39]; however, the underlying mechanisms of the other types remain incompletely elucidated. It is highly likely that additional pathogenic variants and mechanisms of increased vascular permeability remain to be discovered. Diagnostic tests or predictive therapy based on angioedema type await future confirmation or elucidation of the underlying mechanisms.

**Table 1:** Classification of the types of HAE-nC1-INH without hives based on HAE-linked gene variants

HAE type	Gene	Mutation	Amino acid change	First description of HAE-linked gene mutation	Methods used
HAE-FXII	*F12*	c.983C > A	p.Thr328Lys	Dewald and Bork 2006 [29]	Candidate gene, Sanger sequencing, linkage analysis
HAE-FXII	*F12*	c.983C > G	p.Thr328Arg	Dewald and Bork 2006 [29]	Candidate gene, Sanger sequencing, linkage analysis
HAE-FXII	*F12*	c.971_1018 + 24del72	indel	Bork et al. 2011 [40, 41]	Sanger sequencing, linkage analysis
HAE-FXII	*F12*	c.892_909dup	p.Pro298_Pro303dup	Kiss et al. 2013 [41]	Sanger sequencing, linkage analysis
HAE-PLG	*PLG*	c.988A > G	p.Lys330Glu	Bork et al. 2018 [30]	WES, linkage analysis, Sanger sequencing
HAE-ANGPT1	*ANGPT1*	c.807G > T	p.Ala119Ser	Bafunno et al. 2018 [31]	WES, linkage analysis
HAE-KNG1	*KNG1*	c.1136T > A	p.Met379Lys	Bork et al. 2019 [32]	WES, linkage analysis, Sanger sequencing
HAE-Myoferlin	*MYOF*	c.651G > T	p.Arg217Ser	Ariano et al. 2020 [33]	WES, linkage analysis
HAE-HS3OST6	*HS3ST6*	c.430A > T	p.Thr144Ser	Bork et al. 2021 [34]	WES, linkage analysis, Sanger sequencing
HAE-unknown	not identified	n.a	n.a	n.a	n.a

*ANGPT1* angiopoietin-1, *F12* factor XII gene, *FXII* factor XII protein, *HAE* hereditary angioedema, *HS3ST6* heparan sulfate 3-O-sulfotransferase 6 gene, *KNG1* kininogen-1 gene, *MYOF* myoferlin gene, 3-OST-6 3-O-sulfotransferase 6 protein, *PLG* plasminogen gene, *WES* whole exome sequencing, *n.a.* not applicable

Two additional forms of recurrent angioedema without hives need to be considered. These are idiopathic non-mast cell-mediated angioedema (INMA; also called angioedema unknown type [AE-UNK] in the DANCE classification [14] and previously referred to as idiopathic non-histaminergic angioedema [42]) and acquired C1INH deficiency. INMA closely resembles HAE-nC1INH but is distinguished by both the lack of a family history and the lack of a known pathogenic variant. Given the variable penetrance of HAE-nC1INH, patients currently classified with INMA may ultimately be shown to have HAE-nC1INH based on discovery of new pathogenic variants or identification of affected family members. Acquired C1INH deficiency often closely resembles HAE-C1INH, except that patients develop angioedema later in life (usually after the age of 40), lack a positive family history of angioedema, often show low plasma levels of C1q, and frequently have (or will develop) an underlying disease (especially lymphoproliferative malignancies, monoclonal gammopathies, or autoimmune diseases such as systemic lupus erythematosus) or C1INH autoantibodies.

### Clinical Features that May Differentiate HAE-nC1INH from other Forms of Angioedema

**Fig. 1:**
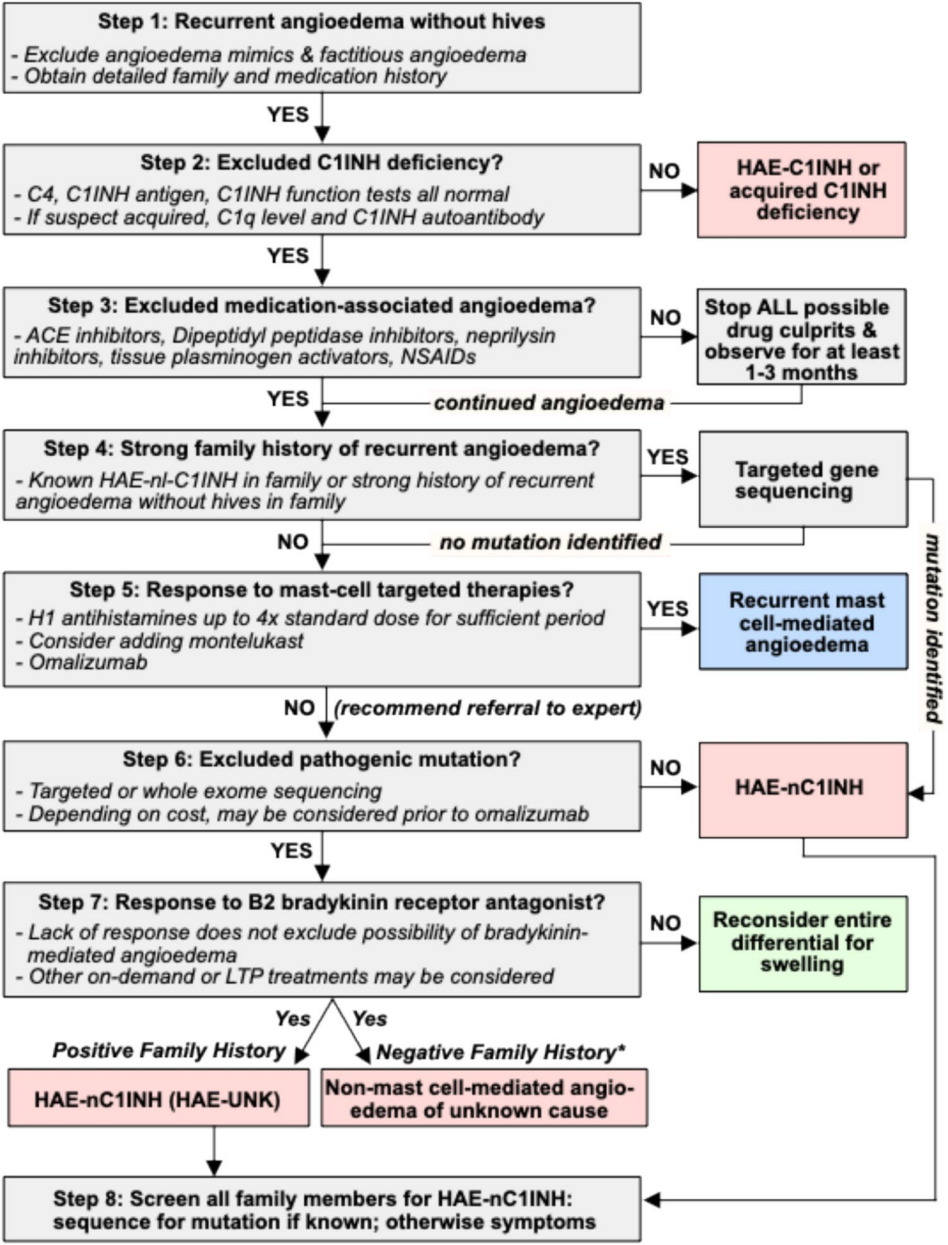
Algorithm for diagnosis of recurrent angioedema with normal C1 inhibitor. *Family history may be an unreliable marker of HAE as detailed in the text

Table 2 summarizes the clinical features distinguishing mast cell–mediated angioedema, HAE-C1INH, HAE-nC1INH, and INMA. The diagnostic algorithm (see Fig. 1 and below) utilizes these features in evaluating patients with recurrent angioedema without hives.

**Table 2:** Non-exhaustive observed characteristics of mast cell–mediated angioedema, different types of hereditary angioedema and INMA

	Disease											
	AE-MC	HAE-C1INH	HAE-nC1INH							New Possible HAE-nC1INH		INMA
			HAE-FXII	HAE-PLG	HAE-KNG1	HAE-ANGPT1	HAE-MYOF	HAE-HS3OST6	HAE-UNK	HAE-CPN1	HAE-DAB2IP	
Family history	-	75%	67–92%	> 90%	+	+	+	+	+	+	+	-
Sex (female/male ratio)	2:1 to 1:2	1:1	10–13:1	3:1	4:0	5:1	3:0	Female only	1:3 to 5:1	2.4:1	1:1	2:1
Typical age at symptom onset	20–60 yrs	10–20 yrs	20–26 yrs (1–65 yrs)	20 yrs	35 yrs	> 20	10–20	12–20	25–30	12–47	Infant–20 yr	30–50 yrs
Location of attacks Extremities Face Upper airways Tongue GI tract Genitals	+	+ +				+ +		+		+ +	+ +	+ +
	+ +	+	+	+	+ +	+ +	+ +	+ +	+ +		+	+ +
	+	+	+	+			+			+	+	
	+	+	+	+ + +	+ +	+ +	+ +		+ +	±		+
	+	+ +	+ +			+ +		+ +		+ +	+ +	+
	+	+									+	
Frequency of attacks	Variable; average of 6/year	Average of twice per month	May be long symptom-free intervals			Infrequent					Twice weekly to every other month	
Duration of attacks	Rapid onset with resolution within 1–2 days typically	Slow onset and resolution, usually 3–4 days					12 h				3 days	
Triggering factors Foods Insect Estrogens Mechanical trauma	+ +	+							-			
	+ +	+							-			
	-	+ +	+ +	+	+	UNK	UNK	+	+	+	+	+
	+	+ +	+ +					+ +	+	+	+	
Pregnancy impacting attacks		+ +	+ +				+ +	+ +	-			+
Erythema marginatum prodrome	-	+ +										-
Other sign			Bleed or hemorrhage into skin				Nailfold capillary ectasia					
Accompanied with hives (except for incidental comorbidity of hives)	+ +	-	-	-	-	-	-	-	-	+	+	-
Response to mast cell treatment	+ +	-	-	-	-	-	-	-	-	±	-	-

+ : rare, + + common

#### Prevalence

Although angioedema is common, with ~ 7.4% of the population estimated to have experienced at least one episode, HAE is rare (Table 3). HAE due to C1INH deficiency (HAE-C1INH) affects around 1 in 50,000 [43, 44]. Indeed, population-based epidemiological investigations showed from 1.07 per 100,000 up to 1.56 per 100,000 [44–50].

**Table 3:** Estimated prevalence for HAE

Angioedema type	Estimate of prevalence	Comments	Reference
HAE-C1INH	1.10 per 100,000/1 in 91,162	Spain	[51]
	1.50 per 100,000/1 in 66,597	Norway	[46]
	1.38 per 100,000/1 in 72,671	Denmark	[47]
	1.56 per 100,000/1 in 64,028	Sweden	[48]
	1.51 per 100,000/1 in 66,284	Italy	[49]
	1.07 per 100,000/1 in 93,235	Greece	[50]
HAEnC1INH (all types)	1 in 100,000	Estimate from large German centre	[52]
	1 in 248–268,000	US physicians survey	[53]
HAE-FXII, (4 known variants)	561 cases/281 families reported 1 in 150,000	Founder effect results in geographic variation in prevalence	[54]
HAE- PLG p.Lys330Glu	172 cases/45 families reported	Various ethnicities	[54]
HAE_ANGPT1 p.Ala119Ser	4 cases/2 families reported	Italian	[54]
HAE-KNG1 (2 variants)	14 cases/7 families	German/ Italian	https://www.omim.org/entry/619363?search=HAE6&highlight=hae6
HAE-MYOF p.Arg217Ser	3 cases/1 family	Italian	https://www.omim.org/entry/619366?search=HAE7&highlight=hae7, accessed 9 July 2023
HAE-HS3ST6 p.Thr144Ser	3 cases, 1 family	German	https://www.omim.org/entry/619367, accessed 9 July 23
HAE-CPN	15 cases, 4 families	France	[36]
HAE-DAB2IP	7 cases, 1 family	Argentina	[35]

The prevalence of HAE-nC1INH is difficult to estimate given the uncertainty of diagnosis but may be even rarer. One large German center estimates a prevalence of 1 in 100,000 for HAE-nC1INH, 1 in 150,000 for HAE-UNK, and 1 in 400,000 for HAE-FXII [52]. A recent US physician survey estimated a prevalence of 1 in 248,000–268,000 [53]. Geographic variability in prevalence is likely as follows: the most common genetic type, HAE-FXII p.Thr328Lys, is believed to originate from a founder pathogenic variant [55]. Other variants in *F12* are limited to single families [7]. Overall, the worldwide reported number of cases is low, totaling 782 patients in 343 families for all genetically identified HAE-nC1INH types (Table 3) [54]. It is likely, however, that many patients remain to be diagnosed and thus the true prevalence of HAE-nC1INH is unknown.

#### Clinical Features Distinguishing HAE-nC1INH from Mast Cell–Mediated Angioedema

Unlike the vast majority of mast cell–mediated angioedema, HAE-nC1INH patients have either a family history of recurrent angioedema or a genetic pathogenic variant in one of the genes known to be associated with it. Attack trajectory and location are important distinguishing features. HAE-nC1INH attacks tend to progress more slowly, last longer, and are more likely to involve the abdomen or require intubation compared to mast cell–mediated angioedema [56, 57]. Treatment response is another important distinguishing feature between the two types of angioedema. Unlike mast cell–mediated angioedema, patients with HAE-nC1INH show no response to high-dose H1 antihistamines, corticosteroids, epinephrine, leukotriene receptor antagonists, or omalizumab but may respond to KKS targeted treatments such as C1INH concentrates, bradykinin B2 receptor antagonists, or plasma kallikrein inhibitors (see treatment section below). Fibrinolytic inhibitors such as tranexamic acid or anti-estrogens, such as danazol or some progestins, may also be effective in HAE-nC1INH. Mast cell–mediated angioedema is the most common diagnosis in patients with recurrent angioedema, underscoring the urgent need for a reliable biomarker to distinguish these diagnoses.

#### Clinical Features Distinguishing HAE-nC1INH from HAE-C1INH

While the clinical features of HAE-nC1INH in an individual patient may not be distinguishable from HAE-C1INH, there are some general differences between the groups. HAE-nC1INH tends to be characterized by lower penetrance of disease expression, later age at onset of swelling, a female preponderance, and in some subtypes a more impactful role of estrogens on disease expression [58]. HAE-nC1INH attack frequency may also be variable with long periods between attacks. Other features of HAE-nC1INH include a lack of reports of erythema marginatum as a prodrome and bruising or hemorrhaging into skin just prior to swelling [59, 60]. There appears to be a greater prevalence of face, tongue, and throat swellings with fewer abdominal attacks and fewer simultaneous swellings at multiple sites when compared with HAE-C1INH. There is a lack of evidence-based data regarding treatment response in HAE-nC1INH; however, there are observations made from case series and reports. Some HAE-nC1INH patients only have symptoms when exposed to high levels of estrogens, and discontinuing estrogen-containing medications may be an effective measure. HAE-nC1INH is also more likely to show beneficial prophylactic responses to treatment with tranexamic acid. For management of HAE-nC1INH attacks, treatment with a plasma-derived C1INH concentrate, bradykinin B2 receptor antagonist (icatibant), or plasma kallikrein inhibitor (ecallantide) is generally effective.

#### Specific Clinical Findings in Individual HAE-nC1INH Subtypes

To date, pathogenic variations in six different genes have been associated with HAE-nC1INH [61]. These are rare types of HAE with pathologic variation in one of the genes having been described in single multigenerational families in some cases, so only limited clinical descriptions are available.

##### HAE-FXII

The best understood subtype is HAE–FXII with the majority of patients with the p.Thr328Lys pathogenic variant reported from Brazil, France, Germany, and Spain. Although autosomal dominant in inheritance, there is incomplete penetrance with more females affected. The average age of symptom onset is 20 years (range 1 to 65 years). Estrogen is a very important trigger in the majority of women with this pathogenic variant. Women were not infrequently present for their initial evaluation of angioedema during pregnancy or when taking an estrogen containing contraceptive pill. Patients with HAE–FXII may experience swelling at a variety of sites including but not limited to extremities, abdominal, tongue, laryngeal, face, and lips. There are several reports of bleeding into the skin with bruising or skin hemorrhaging reported to precede or accompany swellings. Patients with this form may have long symptom-free intervals. Positive response to C1INH concentrates and icatibant for management of attacks is reported.

##### HAE-PLG

The plasminogen pathogenic variant has been described in various European countries, especially in Germany and France, as well as in Japan, Hungary, and the USA. Once again, more women appear to be affected, and on average, there is a later age of onset of swellings. Tongue swellings are reportedly frequent, and death from asphyxiation has been reported [30]. Fewer attacks appear to be related to estrogen therapy or pregnancy when compared to HAE–FXII. Tranexamic acid may be particularly effective in this subgroup.

##### HAE-ANGPT1

This pathogenic variant is rare with very few patients described. Attacks are reported to affect the face, lips, mouth, and abdomen predominantly and appear to be relatively infrequent. Trigger factors have been reported in a minority of attacks.

##### HAE-KNG1

This pathogenic variant has been described in seven families, consisting of 14 individuals. They had had a later age of symptom with a range from 17 to 55 years. Swellings described involved face, tongue, hands, feet, and abdomen.

##### HAE-MYOF

This pathogenic variant has been described in an Italian family. Three of four carriers were described as having symptoms which began in the second decade and mainly involved the face, lips, and oral mucosa. The mother reported a single attack involving the airway. Attacks had an average duration of only around 12 h. Menstruation and warm weather were identified as triggers in these patients.

##### HAE-HS3OST6

This pathogenic variant has been described in one multigenerational family, and all affected individuals have been female, with an age of onset between 12 and 20 years. Attack frequency is reported as variable, between 1.2 and 36 per year. Attack locations described includes the face, extremities, abdomen, larynx, and tongue, with a report of worsening with the use of estrogen therapy.

##### HAE-Unknown

A proportion of patients with a phenotype indicative of HAE-nC1INH (recurrent angioedema that is not mast cell-mediated, normal C1INH function, and a positive family history of angioedema) do not have an identified pathogenic variant. They are classified into the group HAE of unknown cause (HAE-UNK) [52, 62, 63]. In HAE-UNK, angioedema typically first occurs in early adulthood. The angioedema most often involves the face and tongue and less frequently the gastrointestinal tract. Females are more frequently affected.

#### Specific Clinical Findings in Newly Described Hereditary Angioedema with Hives

Since the Symposium meeting, two reports have appeared associating additional mutations with hereditary recurrent angioedema. Interestingly, the clinical phenotype for both of these mutations appears to also include hives as well as angioedema. Further studies will be necessary before we know whether these represent HAE-nC1INH or familial urticaria.

##### HAE-CPN

A familial association between recurrent angioedema and CPN1 deficiency was first reported by Mathews et al. in 1978 prior to the recognition of HAE-nC1INH [64]. Vincent et al. recently reported four unrelated families with angioedema affecting their face, abdomen, and larynx as well as CPN deficiency [36]. In both reports, patients presented with hives as well as angioedema. Symptoms were associated with estrogens in several of the families [36]. In several patients, elevated histamine levels were detected during attacks [64].

##### HAE-DAB2IP

This pathogenic variant was found in seven members (three female and four male) of a three-generation family from Argentina in whom affected members suffered from recurrent angioedema. Four of these seven patients also had recurrent hives involving multiple locations [35]. The angioedema was worsened by estrogens.

#### Clinical Features Distinguishing HAE-nC1INH from INMA

The major differentiating features between HAE-nC1INH and INMA are the lack of a family history of swelling and the absence of known genetic pathogenic variants.

#### Limitations of the Clinical Characteristics

There are limitations in diagnosing HAE-nC1INH on clinical signs and symptoms alone. Symptoms may show inter- and intra-individual variability even within a family with the same pathogenic variant. Inclusion of family history as a required criterion for HAE proves problematic. Family history may be an unreliable or inaccurate marker of a hereditary angioedema condition in clinical practice for several reasons, including recall bias; unknown due to estrangement, adoption, or paternal discrepancy; the possibility of de novo mutations (extrapolating from HAE-C1INH); and variable penetrance resulting in phenotypic variation or asymptomatic carriers [7]. Therefore, the presence of a family history of angioedema may be considered strongly supportive of an HAE diagnosis but based on current knowledge and practical limitations cannot be rigorously defended as a requirement for diagnosing HAE conditions. While hives are not found as part of the symptom complex of HAE (with the possible exceptions of HAE-CPN and HAE-DAB2IP), some patients may experience hives as a separate entity. While mast cell–mediated angioedema does not typically involve the gastrointestinal tract, abdominal symptoms are common; imaging showing edema of the bowel wall is critical for distinguishing true abdominal angioedema. Triggers typically reported for HAE attacks such as stress, physical trauma, infection, or estrogens may trigger other forms of angioedema as well. Certain drugs may initiate or worsen symptoms of HAE. Assessment of therapeutic response to conventional therapy is not always informative, as angioedema may resolve spontaneously.

Very limited data are available on disease-specific biomarkers in HAE-nC1INH beyond the identified pathogenic variants that affect only a subset of patients. Several potential biomarkers have been proposed; however, none are yet fully validated and available for routine clinical practice [65–67].

### Diagnostic Algorithm for Diagnosis of HAE-nC1INH

The diagnosis of HAE should be considered in all patients with recurrent angioedema without hives. Proper diagnosis utilizes four modes of investigation: (1) clinical presentation (phenotype), (2) pathomechanism, (3) biomarkers, and (4) genetics. The diagnostic process is complicated by the reality that in clinical practice, many angioedema patients will have an unknown pathomechanism while validated biomarkers and genetic studies are either unavailable or uninformative. Thus, a combination of all the above parameters is highly desired but often not attainable. In the absence of helpful laboratory data, clinicians must assess the patients’ responses over time to empiric treatment with medications targeting potential pathogenic pathways. This strategy can also be challenging as it requires prolonged patient engagement in a systematic stepwise treatment approach and may lack robust objective methods to assess efficacy. The algorithm below and Fig. 1 summarize the recommended approach for evaluating these patients. This approach may need to be further adapted according to the available expertise, testing facilities and resources. Patients presenting to an emergency department with an angioedema attack of unknown cause should be referred to a physician familiar with angioedema who can undertake this evaluation.

#### Step 1. Is the Diagnosis Tenable?

Confirm a clinical history of documented recurrent angioedema without hives. Photos and laryngoscopic and imaging evidence of angioedema are essential to differentiate true angioedema from factitious angioedema or patients who mistakenly believe they have angioedema based on non-angioedematous symptoms, both of which are common in patients (mis)diagnosed with HAE-nC1INH (Table 4) [68, 69]. In the case of predominant gastrointestinal attacks, obtaining abdominal imaging during an attack can be useful to evaluate for bowel wall edema and intraperitoneal fluid. A detailed family history and medication history should be obtained in all patients with recurrent angioedema.

**Table 4:** Differential diagnosis of angioedema and angioedema mimics

Non-mast cell mediated	HAE-C1INH, HAE-nC1INH, fibrinolytic therapy associated, kininase associated (ACE, DPP4, or neprilysin inhibitors), INMA
Mast cell mediated	Direct mast cell release, chronic urticaria, mast cell activation syndrome
Other differentials (mimics)	Lymphedema; hypoalbuminemia; peripheral edema (right heart failure, use of calcium channel blocker, idiopathic oedema); thyroid disease (Grave’s disease, hypothyroidism [myxedema]); venous obstruction (e.g. superior vena cava syndrome); infiltrative diseases (amyloidosis, IgG4-related disease, granulomas, malignancies, Sjögren syndrome); systemic capillary leak syndrome (Clarkson syndrome); eosinophilic disorders (episodic angioedema with eosinophilia [Gleich’s syndrome], nonepisodic angioedema with eosinophilia, eosinophilic cellulitis [Well’s syndrome]); Melkersson-Rosenthal syndrome, orofacial granulomatosis; Morbihan disease Abdominal symptoms: acute abdomen; acute intermittent porphyria; endometriosis, familial mediterranean fever

#### Step 2. Exclude C1INH Deficiency

Measure C4, C1INH antigen, and C1INH function (if available), even if the patient is taking a medication that may cause angioedema. If acquired C1INH deficiency is suspected based on age of symptom onset, C1q level and anti-C1INH antibodies should be measured. Sequencing of *SERPING1* is not routinely required, but can be helpful to distinguish acquired C1INH deficiency from HAE-C1INH, especially in patients aged > 40 years.

#### Step 3. Exclude Medication-Associated Angioedema

Stop the suspected medication and assess response, which may take 1–2 months or longer depending on the frequency of the episodes.

#### Step 4. Genetic Screening for Pathogenic Variants

A detailed family history for evidence of recurrent angioedema or a diagnosis of HAE in other family members is essential in all patients with recurrent angioedema. If the patient has a strong family history of angioedema or known relatives with HAE-nC1INH, then consider targeted gene sequencing (see step 6 below).

#### Step 5. Exclusion of Mast Cell–Mediated Angioedema (AE-MC)

Evaluate for mast cell–mediated angioedema based on clinical symptoms (Table 2) as well as response to mast cell–directed therapy. This must include an adequate trial of daily second-generation H1 antagonist (fourfold times the standard dose assuming lower doses fail to prevent angioedema attacks) for a duration sufficient to clearly determine the treatment effect. When H1 antihistamines fail as a single treatment, daily montelukast is added unless there is a contraindication [16, 70, 71]. If unresponsive to high-dose antihistamines plus montelukast, a course of omalizumab (4–6 months) is warranted. Most cases of AE-MC respond well to omalizumab [17]. Response to any of these medications suggests the diagnosis of mast cell–mediated angioedema. If no response, we strongly recommend seeking assistance of an angioedema expert prior to proceeding with the next steps.

#### Step 6. If Unresponsive to Step 5, Targeted Gene Sequencing

Targeted sequencing (next-generation sequencing [NGS] or Sanger sequencing), for known HAE pathogenic variants is recommended if accessible [72, 73]. If a pathogenic variant is found, the diagnosis of HAE-nC1INH is established. A novel variant in one of the known genes needs to be considered a variant of unknown significance (VUS) and not a cause of HAE-nC1INH until confirmed by further research [74]. Depending on the availability and cost of sequencing, this step may best be undertaken prior to a course of omalizumab (step 5). If sequencing is not available, the clinician can proceed with step 7.

#### Step 7. Consider a Short Course of a Bradykinin B2 Receptor Antagonist

In the absence of a known pathogenic variant, patients with a reliable positive family history of angioedema are classified as HAE-UNK while those without a family history are classified as INMA. For both, a prompt and durable response to a bradykinin B2 receptor antagonist or another approved HAE on-demand treatment (if available) early in an attack is evidence helping to support the diagnosis of bradykinin-mediated angioedema. However, lack of such a response does not necessarily exclude HAE. A short course of a second on-demand HAE medication or a long-term prophylactic treatment such as tranexamic acid may be considered.

#### Step 8. Screening of Family Members

When a diagnosis of any type of HAE is made, all related family members should be screened by clinical history for HAE, including whether female family members encountered swelling during states of elevated estrogen. Targeted genetic screening should be done on family members, irrespective of whether or not they have experienced angioedema, when HAE-nC1INH with a known pathogenic variant is identified [75].

Not all physicians will have access to the testing suggested above. In addition, some of the steps in the algorithm can lead to inconclusive results. Therefore, we recommend that patients being considered for a diagnosis of HAE-nC1INH or INMA have a physician experienced in the diagnosis with access to modern testing be involved in the case.

## Section 2: Genomics, Pathophysiology, Biomarkers

### Genomics

Pathogenic variants of the coagulation factor XII gene (*F12*) were the first genetic alterations identified in patients with HAE-nC1INH [29]. Thereafter, the expanded use of genomic technologies identified pathogenic variants in genes, namely, *ANGPT1*, *PLG*, *KNG1*, *MYOF*, and *HS3ST6* [76]. All of these variants are transmitted as autosomal dominant traits with incomplete penetrance.

*F12* pathogenic variants are estimated to account for 83.4% of families diagnosed with HAE-nC1INH with 99.4% carrying the missense variant c.983C > A (p.Thr328Lys). Another missense variant, the c.983C > G (p.Thr328Arg), the deletion c.971_1018 + 24del72, and the duplication variant c.892_909dup have also been found associated with HAE-nC1INH. All these variants occur in exon 9 of *F12* gene [54]. The exon 9 encodes a highly glycosylated proline-rich region of the protein, adjacent to the cleavage site upon activation leading thus to an increased activation of FXIIa via activation by plasmin [37, 39, 55].

*F12* pathogenic variants were most frequently identified in Brazil, France, Spain, Turkey, and Germany, with a smaller number identified in many other countries [54]. However, they have not been identified in Japanese families, suggesting an ethnic difference of these variants [77].

HAE-FXII is inherited with incomplete penetrance, which is very low in male patients (male/female = 1:10) [52]. The higher female predominance of HAE-FXII is mostly explained by the recent observation that during early embryogenesis, HAE-nC1INH-linked variants favor a selection leading to a loss of 20–25% of male embryos [78]. Moreover, the higher female predominance as well as the estrogen dependency of the clinical phenotype of HAE-FXII might be also attributed to the positive regulation of the expression of the *F12* gene by estrogens due to the presence of estrogen-responsive elements in its promoter region [79].

The next widely identified pathogenic variant of HAE-nC1INH globally is c.988A > G in the *PLG* gene located on chromosome 6 and encoding plasminogen protein [30]. This variant presents with incomplete penetrance and a male/female ratio of 1:3. (3) Located in exon 9, it leads to the missense pathogenic variant p.Lys330Glu in the kringle 3 domain of the plasminogen protein [80].

The *ANGPT1* gene c.355G > T;p.Ala119Ser variant has been found co-segregated with HAE-nC1INH in four patients from one Italian family [31]. Two more variants of *ANGPT1*, p.Ala8Val and p.Gln370His, have been detected in HAE-nC1INH families, the pathogenicity of which remains to be confirmed [81].

Another pathogenic variant has been identified in 14 cases within 7 families including 6 affected members of a three-generation German family with HAE-nC1INH [32]. This is a missense variant c.1136T > A;p.Met379Lys in the *KNG1* gene located on chromosome 3 and encoding high molecular–weight kininogen (HK) and low molecular–weight kininogen (LK) by alternative splicing. The pathogenic variant occurs in domain 4, close to the cleavage site for kinin production and, therefore, could alter that process. However, the mechanism remains to be confirmed.

The exon 7 missense c.651G > T;p.Arg217Ser variant of the *MYOF* gene located on chromosome 10 was identified in three HAE-nC1INH patients from one Italian family, one more member of which was an angioedema-free carrier of the variant [33]. Myoferlin is a multifunctional protein expressed on endothelial and other cells. The p.Arg217Ser variant is located in the C2B domain of the protein, and existing evidence indicates that it is a gain-of-function variant effectively translocating the vascular endothelial growth factor (VEGF) receptor 2 (VEGFR2) to the plasma membrane.

A variant associated with HAE-nC1INH was detected in a single German family, and it is a missense pathogenic variant in exon 2 of the *HS3ST6* gene, c.430A > T; p.Thr144Ser located in chromosome 16, which encodes the HS-glucosamine 3-O-sulfotransferase 6 (3-OST-6) protein [34].

It should be recognized that many of the mutations associated with HAE-nC1INH have been reported in only a very limited number of patients (see prevalence section above). Furthermore, the pathogenic mechanisms by which mutations cause angioedema (see below) have only been clearly delineated for HAE-FXII although there are now reasonable mechanisms demonstrated for HAE-PLG and HAE-KNG. The nature of the association between the other mutations and the hereditary angioedema phenotype remain to be confirmed.

Pathologic variants have also been identified in the newly described cases of hereditary angioedema plus hives. Three variants of CPN were described that associated with a phenotype of recurrent angioedema and hives. The three variants c.533G > A, c.582A > G, and c.734C > T were identified in the *CPN1* gene encoding the catalytic subunit of carboxypeptidase N, also known as kininase I, and located in chromosome 10 [36].

A variant of *DAB2IP* was described that was associated with recurrent angioedema and occasional hives in a single Argentinian family. The pathogenic missense variant c.715G > A;p.Asp239Asn, exon 6 of the *DAB2IP* gene, is located in chromosome 9 [35].

### Pathophysiology

#### HAE-FXII

In 2000, patients with strong family histories of angioedema were reported; most were women, and attacks of swelling were related to the use of estrogen [27, 28], but C1INH was normal. Mutations of *F12* were responsible, the most common being replacement of a threonine by arginine or lysine [29] which disrupted an *O*-glycosylation site [37, 82]. The inheritance pattern was autosomal dominant so that half the FXII is mutated, while half is normal. Addition of low concentrations FXII activators to HAE-FXII plasma leads to overproduction of bradykinin. Site-directed mutagenesis of Thr_309_ to alanine, which also disrupted the O-glycosylation site, did not facilitate FXII activation, suggesting that the specific presence of lysine or arginine was important. This was demonstrated by a rapid proteolytic cleavage at the Lys/Arg_309_-Thr_310_ variant bond, with production of a smaller FXII species, termed δFXII, not yet activated [39]. Plasmin was first shown to be responsible for this proteolytic step [83] and later activated factor XI and thrombin were found to act similarly [39]. A second cleavage by plasma kallikrein (or plasmin) at the usual Arg_353_-Val_354_ bond of δFXII occurred at least 15-fold more rapidly than for non-mutant FXII [39].

The consequence is KKS overactivation, with bradykinin production, and together, the rate is sufficiently fast to overwhelm control rate by C1INH [84]. Because it lacks a heavy chain, δFXII does not participate in contact activation: angioedema in patients with FXII-Lys/Arg_309_ results from solution-phase KKS activation.

#### HAE-PLG

Among the various forms of HAE-nC1INH, the second most frequently encountered appears to be a mutation in the plasminogen molecule (HAE-PLG). The specific mutation commonly seen is p.Lys330Glu located in Kringle 3 [30]. The transmission is a typical autosomal dominant, yet here too, there is a predilection for expression in women but not as skewed as is the *F12* mutation. Angioedema might be the result of interaction of plasmin (after conversion of plasminogen to plasmin) with any of the components of the bradykinin-forming cascade to augment bradykinin formation. When each of these steps was evaluated, only cleavage of HK by mutant plasmin [38] far exceeded what was observed with unmutated plasmin. The presence of either FXII or plasma kallikrein was irrelevant; thus, mutant plasmin resembled activated plasma kallikrein in its ability to generate bradykinin from HK. However, mutant plasmin cleaved LK even more readily [38]. In contrast to tissue kallikrein, which preferentially cleaves LK, mutant plasmin did not yield Lys-bradykinin but produced bradykinin directly. Since there is three times more LK compared to HK in human plasma, one can surmise that most bradykinin formation in HAE-PLG may be generated from LK [85]. Mutant plasminogen-Glu_330_ appears to liberate bradykinin when incubated with HK, without being converted to plasmin [86]. This suggests that mutant zymogen plasminogen has significant activity. The mutant plasminogen has also been shown to be more easily activated to plasmin compared to wild-type plasminogen [80]. It would be of particular interest to compare mutant plasminogen vs mutant plasmin for enzymatic activity on both kininogens.

#### HAE-ANGPT1

The *ANGPT1* p.Ala119Ser variant has been reported in patients with HAE-nC1INH [87]. Angiopoietin-1 is a secreted protein that binds to its receptor tunica internal endothelial cell kinase 2 (TIE2), a receptor primarily expressed in growing vascular endothelial cells. The multimeric angiopoietin-1-TIE2 signaling axis inhibits the effects of several permeability factors, including VEGF and bradykinin, and contributes to the maintenance of the endothelial barrier function. The mutated angiopoietin-1 p.Ala119Ser forms a reduced amount of multimers and shows reduced binding capability to its receptor TIE2 on endothelial cells [88]. *ANGPT1* mutation leads to increased vascular permeability and angioedema with a loss of function mechanism.

#### HAE-MYOF

*MYOF* variant p.Arg217Ser has been reported as a cause of HAE-nC1INH in an Italian family (mother and two daughters with history of recurrent angioedema without urticaria) [33]. Myoferlin is a transmembrane protein located at the plasma membrane of endothelial cells. It modulates VEGF signal transduction by preventing VEGFR2 degradation. The p.Arg217Ser variant allows for a higher ability of the mutant protein to localize VEGFR-2 to the plasma membrane in response to VEGF stimuli. The p.Arg217Ser variant is hypothesized to cause angioedema by an overwhelming activation of VEGF-mediated intracellular signaling. Therefore, the p.Arg217Ser variant influencing vascular permeability regulation results in a gain-of-function variant.

#### HAE-KNG1

Data is emerging about how *KNG1* variants may cause angioedema. *KNG1* encodes both HK and LK via alternative splicing. Plasma kallikrein cleaves HK between Lys_380_ and Arg_381_, releasing bradykinin. Tissue kallikrein cleaves LK between Met_379_ and Lys_380_, releasing Lys-bradykinin, another high-affinity B2 receptor agonist [89].

*KNG1* c.1136T > A causes an amino acid change of methionine to lysine at the 379 position, which is at the N-terminal cleavage site of the bradykinin sequence of both HK and LK [32]. This variant increases HK and LK susceptibility to cleavage by plasmin, resulting in release of Lys-bradykinin [90].

*KNG1* c.1720C > G causes an amino acid change of proline to alanine at the 574 position [91] in domain 6 of HK, which facilitates binding of HK to factor XI and prekallikrein. It appears to cause recurrent angioedema in patients who are also heterozygous for *ACE* c.1459C > T.

#### HAE-HS3OST6

An additional variant causing HAE-nC1INH has been reported in the *HS3ST6* gene, p.Thr144Ser [34]. It encodes an enzyme, 3-OST-6, implicated in the last step of heparan sulfate (HS) biosynthesis. The reported mutation is thought to interfere in the ability of the HS3ST6 to transfer sulfur groups to the 3-OH position of HS, resulting in incomplete HS biosynthesis. The different structure of surface proteoglycans, due to mutation, is likely to affect endothelial surface interactions of key players in angioedema formation. The hypothesis is that the reduced HK endocytosis allows for HK cleavage and increased bradykinin production.

#### HAE-UNK

In the recent years, there was an important improvement in the understanding of the pathogenesis of HAE-nC1INH. Variants of the *F12*, *ANGPT1*, *PLG*, *KNG1*, *MYOF*, and *HS3ST6* genes have been detected [13]. Nevertheless, in a large portion of patients with hereditary angioedema, no causative variants have been described (HAE-UNK), and the pathophysiology of the disease remains unknown (1). In the HAE-UNK form, potential pathogenic explanations require further investigation. A search for HAE-nC1INH-linked mutations in patients with solitary recurrent angioedema may lead to the detection of patients and families with HAE-nC1INH [7].

#### Newly Described Potential Types of HAE-nC1INH

Recently and after the Symposium, families with recurrent angioedema combined with recurrent urticaria associated with mutations in either *CPN1* or *DAB2IP* genes have been reported [35, 36].

CPN is a peptidase that cleaves C-terminal basic residues from a variety of biologically active peptides and proteins. Low CPN activity could increase the risk of angioedema via a number of mechanisms, including slowing the degradation of kinins, slowing the degradation of anaphylatoxins, or downregulating activation of plasminogen [92].

DAB2IP is a Ras GTPase-activating protein and a cytoplasmic adapter for various signaling pathways. It has promiscuous effects, regulating cell responses to multiple extracellular inputs. One of the proteins it interacts with is the VEGFR2 protein, possibly influencing vascular permeability.

### Biomarkers

A biomarker is a biological observation that substitutes for and ideally predicts a clinically relevant endpoint. Ongoing research is attempting to develop useful and widely available biomarkers that will aid in the diagnosis of HAE-nC1INH [93, 94] (Table 5).

**Table 5:** Biomarkers for the diagnosis of HAE

Biochemical marker	HK cleavage	Kinin split peptides	FXII circulating species	Kinin catabolism enzymology	Acquired PAI2 deficiency	Threshold-stimulated kallikrein activity	Serum glycoprotein 120 cleavage	Cold-induced activation
Application to HAE	HAE-C1INH HAE-nC1INH with impact on KKS activity	HAE-C1INH HAE-nC1INH depending on kinins	HAE-FXII doublet by immunoblot	HAE-C1INH HAE-FXII HAE-CPN	HAE-FXII	bradykinin-mediated HAE	HAE-nC1INH	HAE-nC1INH
Advantage	Estimation of bradykinin production	Estimation of bradykinin production	Half-glycosylated circulating FXII (doublet)^16^	Severity and diagnostic marker	Plasmin-mediated activation of FXII in HAE-FXII patients depletes plasma of PAI2	DXS activates KKS in a dose-dep manner HAE-nC1INH plasma has a lower threshold for zymogen activation	Serum glycoprotein 120 binds to and is cleaved by plasma kallikrein	KKS activation in HAE-nC1INH plasma upon incubation at 4 ºC with kaolin^12^ or DXS.^9^
Performance of testing (ROC)	AUC = 0.93^6^ Specificity: 98% Sensitivity: 54%	Not evaluated	Non evaluated		Non evaluated	AUC > 0.91^8^ Specificity: 80–100% Sensitivity: 86–89%	Non evaluated	Non evaluated
Restriction of analytical application	Quantitative immunoblot^5,6^/EIA^7^	Quantitative mass spectrometry^3^	Spectrophotometric assays		ELISA	Spectrofluorometric assays (fluorogenic substrate: Z-Phe-Arg-AMC)	Quantitative immunoblot	Quantitative immunoblot Spectrophotometric assay
Registration by medicine agencies	In progress *Lacks standardization*^*5,6,7*^	NO	NO	YES for ACE activity	NO	NO	NO	NO
Reference	[95–97]	[98]	[37]		[99]	[86]	[100]	[65, 67]

#### Genetic Biomarkers

Known pathogenic variants of the *F12*, the *ANGPT1*, the *PLG*, the *KNG1*, the *MYOF*, and *HS3ST6* genes are the only diagnostic biomarkers available for the diagnosis of the corresponding types of HAE-nC1INH. Genetic testing for *CPN1* and *DAB2IP* pathogenic variants may also be considered. Testing for these variants when available is recommended for all patients who are suspected to have HAE and have normal C1INH levels and function [13]. Moreover, using these biomarkers, HAE-nC1INH can be diagnosed in patients with long-standing recurrent non-histaminergic angioedema who are solitary in their families as well as in their relatives. However, it should be recognized that many patients suspected to have HAE-nC1INH based on symptoms and clinical course do not have these identified genetic variants [7].

Accumulating evidence indicates that modifier genes could explain the incomplete penetrance and the variable expression of phenotype characterizing HAE, and, therefore, that variants of these genes could be used as prognostic biomarkers [101]. Among them, the most well-studied *F12*‐46C/T polymorphism has been shown that modulates the age of onset of symptoms and the penetrance of the HAE-C1INH [102, 103]. More recently, it has been found that the same polymorphism significantly influences the degree of contact system activation and the clinical severity of the HAE-FXII [104]. Further research in large cohorts of patients most probably would prove the usefulness of genetic biomarkers in the management of HAE-nC1INH.

#### Biochemical Biomarkers

No standardized biochemical markers exist for the diagnosis or follow-up of patients with HAE-nC1INH, with diagnosis currently relying on genetic analysis. Direct determination of bradykinin levels has proven feasible but is technically hindered by the short half-time of bradykinin in plasma [105, 106]. The analysis of stable metabolic peptides applicable to HAE patients has been demonstrated in small-scale pilot studies under highly controlled conditions [98, 107]. Other assays with diagnostic potential have been tested in research laboratories, the most commonly referenced being the relative abundance of HK circulating species resulting from HK cleavage by plasma kallikrein as an indicator of bradykinin release during acute episodes, with both semi-quantitative (immunoblot) [95, 96, 108, 109] and quantitative (immunoassay) [97] settings. This method has been tested in HAE-C1INH patients but also holds diagnostic potential for those HAE-nC1INH subtypes exhibiting increased KKS activation [110].

Plasmatic biomarkers proposed by specialized laboratories include KKS cold activation either fully activated by dextran sulfate [65] or triggered by limiting doses of dextran sulfate [66] or kaolin [67] and cleavage of serum glycoprotein 120 [100]. Furthermore, acquired deficiency of plasminogen activator inhibitor 2 has been reported in HAE-nC1INH with excessive fibrinolysis [99] although it remains an open question as to whether this is a general phenomenon or a specific trait associated with certain HAE-nC1INH subpopulations [67, 100, 111, 112]. FXII-Lys/Arg_309_ variant carriers specifically exhibit a circulating FXII doublet detectable in western blot assays [37]. Finally, increased spontaneous amidase activity has been proposed as a diagnostic tool for bradykinin-mediated angioedema [65] and reduced activity of the bradykinin-catabolic enzymes angiotensin-1 converting enzyme and CPN has been associated with a more severe presentation of the disease in HAE-FXII patients [113]. Detection of low CPN activity should be confirmed by genetic analysis [36].

## Section 3: Treatment of HAE-nC1INH

The treatment strategies used for HAE-nC1INH are similar to those of HAE-C1INH and include the use of on-demand treatment (ODT), short-term prophylaxis (STP), and long-term prophylaxis (LTP) [8–13]. Until now, little is known about the outcomes of treatment in patients with HAE-nC1INH. There are no data from controlled clinical trials, and the limited information available comes from case reports and small case series. Since HAE-nC1INH is a group of conditions that are similar in clinical manifestation but differ in their pathogenesis, differences in treatment responses across the different subtypes of HAE-nC1INH must be expected.

### There is Little Published Information on the Treatment of Patients with HAE-nC1INH

As for HAE-FXII treatments and outcomes, the use of icatibant and plasma-derived C1INH as ODT has been reported to be effective in most of the reported attacks although a significant number also derived little benefit [114–123]. Published information on the use of STP, in HAE-FXII, is limited to less than 10 patients (9 of which were pre-delivery), and all received plasma-derived C1INH, which was interpreted to be effective [117, 123, 124]. As for LTP, progestins, tranexamic acid, androgens, and lanadelumab have been used and reported to be effective in most cases [115, 117, 118, 120, 121, 123, 125–127].

In HAE-PLG, icatibant and plasma-derived C1INH have been used for ODT and reported to be efficacious in most cases [30, 128–131]. Tranexamic acid and androgens are the most used LTP treatments in HAE-PLG as of now, and both were effective in the majority of cases reported [30, 128, 129, 132, 133]. The information on the use and outcomes of any treatments in other types of HAE-nC1INH is very limited, with few published reports [32, 33, 35, 36, 54, 76, 87, 91, 134].

Limited data is available concerning therapy for HAE-CPN1 and HAE-DAB2IP. In the families with CPN1 variants, LTP with tranexamic acid was tried and effective in seven symptomatic subjects and ODT with icatibant was effective in five subjects [36]. In the report describing the DAB2IP variant, four subjects received icatibant for ODT with good responses [35].

### The HAE-nC1INH Consensus Treatment (HCT) Survey Provides Further Information on the Treatment of Patients with HAE-nC1INH

To obtain more information on the outcomes of treatment across the different subtypes of HAE-nC1INH, the HCT survey was developed and distributed to all international angioedema experts invited to the HAEA/HAEi-sponsored 2023 HAE-nC1INH symposium (see supplemental material). This survey provided information on 594 patients with HAE-nC1INH from 15 countries. For those countries with genetic testing available (all with the exception of New Zealand), the total numbers of patients for each HAE-nC1INH subtype were as follows: HAE-FXII, 337; HAE-PLG, 52; HAE-KNG1, 2; HAE-ANGPT1, 1; HAE-MYOF, 6; HAE-HS3OST6, 0; and HAE-UNK, 196. Of note, variants of *CPN* and *DAB2IP* were not identified at the symposium, and therefore, HAE-nC1INH with these mutations were not differentiated from HAE-UNK.

All participating countries had access to and utilized modern ODT, i.e., intravenous plasma-derived or recombinant C1INH, ecallantide, and/or icatibant. STP use was reported in eight countries (Brazil, Israel, France, Japan, Netherlands, New Zealand, Spain, and the US). The predominant LTP treatment was the antifibrinolytic tranexamic acid. Progestins, plasma-derived C1INH, and lanadelumab were used substantially less frequently than antifibrinolytics. Berotralstat and attenuated androgens were used in a relatively small percentage of patients. The use of LTP was reported in all but three participating countries (China, Hungary, Italy). All but one country (New Zealand) had access to a modern prophylactic therapy, i.e., berotralstat, intravenous, or subcutaneous plasma-derived or recombinant C1INH, or lanadelumab. Berotralstat was available in all but five countries (Australia, Brazil, China, Israel, and New Zealand). Intravenous plasma–derived C1INH was available in all but three countries (Israel, Japan, and New Zealand). Subcutaneous plasma–derived C1INH was available in all but four countries (China, France, Israel, and New Zealand). Lanadelumab was available in all countries except New Zealand. The participating global experts ranked their assessments of the effectiveness for ODT and LTP therapies available to them that they used for each type of HAE. Assessments were made using a 5-point Likert scale from worsening to highly effective (see online supplement).

**Fig. 2:**
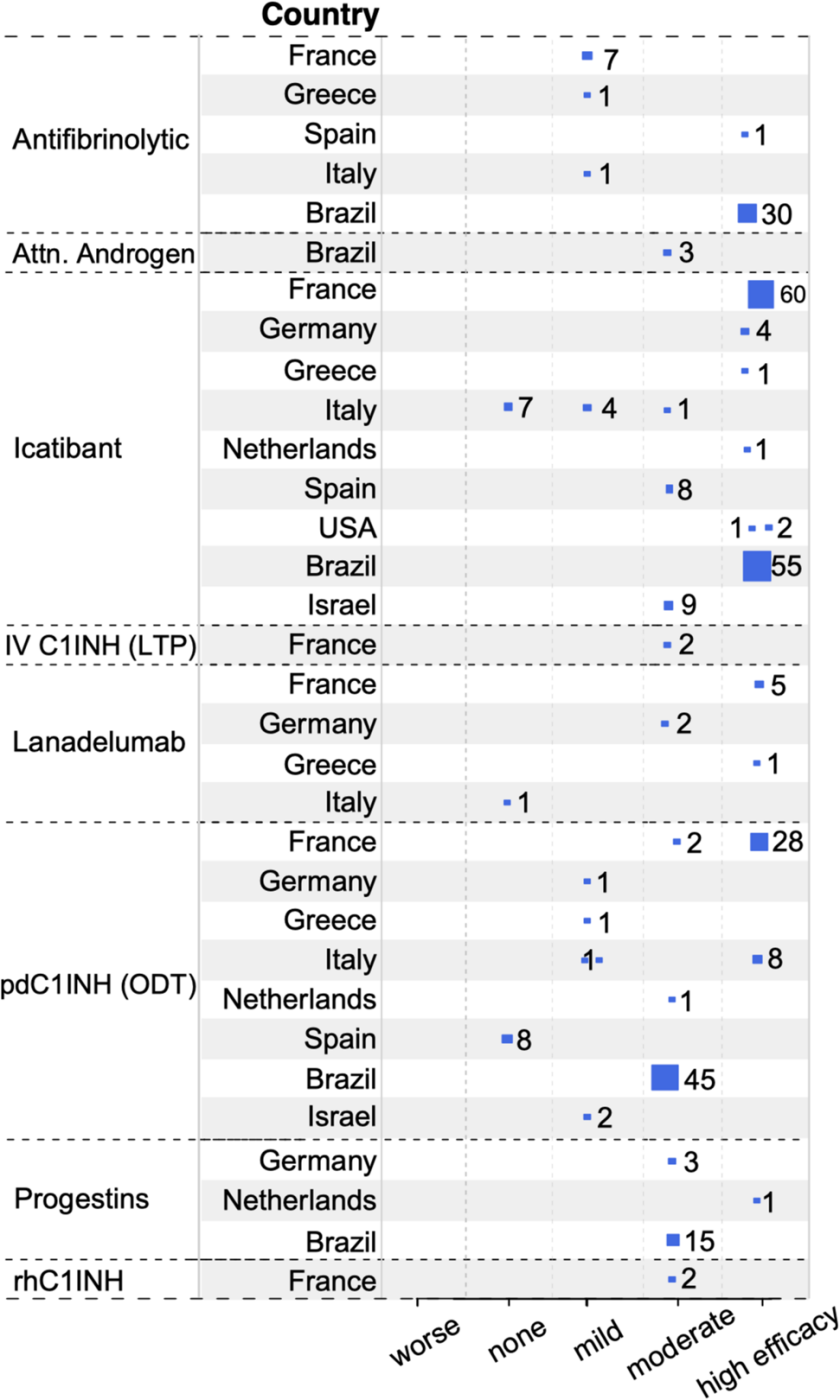
Treatment efficacy for HAE-FXII from the HCT survey. Expert physician ratings of the efficacy of each medication are shown along with the number of HAE-FXII patients treated by each expert. Each blue square represents the report by one expert. The size of the square is proportional to the number of HAE-FXII patients assessed, which is indicated next to it. The larger the square, the more patients showed the indicated response

For HAE-FXII (Fig. 2), the ODT efficacy of icatibant was assessed for 153 subjects and reported as high in 81.0%, moderate in 11.8%, mild in 2.6%, and no change in 4.6%. Plasma-derived C1INH ODT assessments were included for 97 patients, with 37.1% reporting high efficacy, 47.4% moderate efficacy, 6.2% mild efficacy, and 8.3% no change. As LTP therapy, intravenous plasma-derived C1INH was reported as moderately efficacious for two of two patients. Lanadelumab treatment of nine patients was assessed as highly efficacious in six, moderately in two, and non-effective in one. Attenuated androgens, used by three patients from a single center, were assessed as moderately effective. Antifibrinolytics efficacy, in 40 patients, was reported as high in 77.5% and mild in 22.5%. In 19 patients, progestin treatment was assessed as moderately efficacious in 94.7% and highly efficacious in 5.3%. No use of subcutaneous plasma-derived C1INH or berotralstat was reported.

**Fig. 3:**
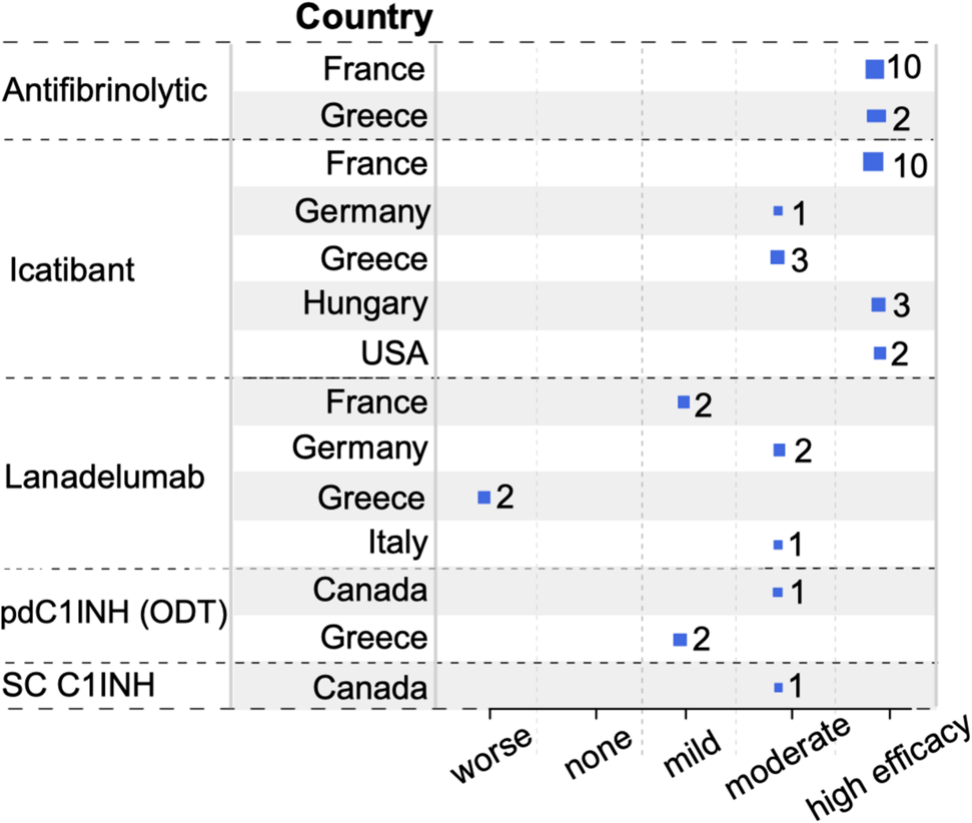
Pretreatment efficacy for HAE-PLG from the HCT survey. Expert physician ratings of the efficacy of each medication are shown along with the number of HAE-PLG patients treated by each expert. Each blue square represents the report by one expert. The size of the square is proportional to the number of HAE-PLG patients assessed, which is indicated next to it. The larger the square, the more patients showed the indicated response

For HAE-PLG (Fig. 3), ODT with icatibant was used by 19 patients and reported as highly and moderately effective in 78.9% and 21.1% of patients, respectively. Plasma-derived C1INH was used in three patients of whom one reported moderate and two mild efficacy. LTP therapy with subcutaneous C1INH was assessed as moderately effective in one of one patient. Lanadelumab treatment of seven patients was assessed as moderately effective in three and as mildly effective in two patients. Two patients were reported to show worsening with treatment. Antifibrinolytics, used in 12 subjects, were assessed as highly efficacious in all cases. Intravenous plasma-derived C1INH, berotralstat, attenuated androgens, and progestin use was not reported.

For HAE-ANGPT1, outcomes of ODTs in three patients were reported, with icatibant as mildly efficacious in two and plasma-derived C1INH as mildly efficacious in one. LTP therapy with antifibrinolytics and progestin each assessed as mildly effective for one patient each. HAE-MYOF treatment outcomes for five patients were reported by one participant, with on demand icatibant and plasma-derived C1INH each mildly effective for one subject and berotralstat moderately effective in three subjects. There were no reports of treatment outcomes for HAE-KNG or HAE-HS3OST6.

**Fig. 4:**
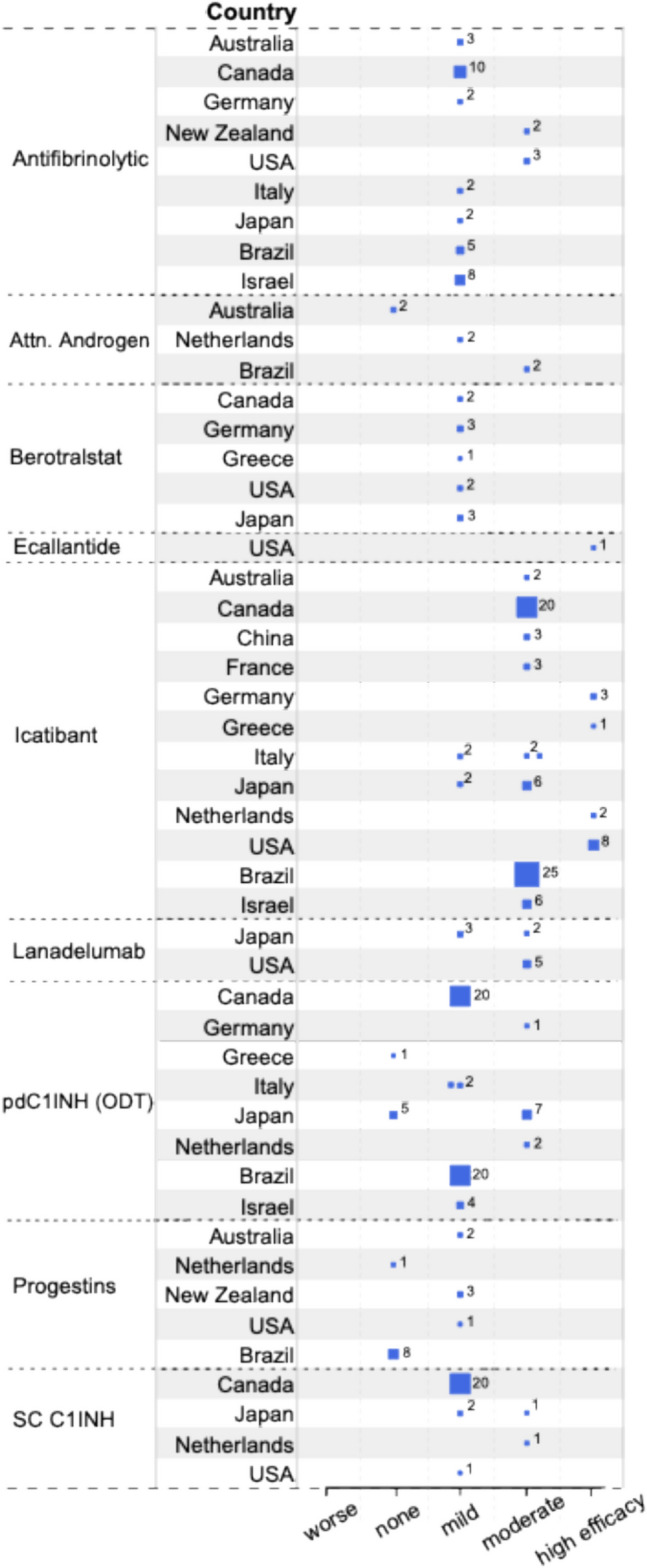
Treatment efficacy for HAE-UNK from the HCT survey. Expert physician ratings of the efficacy of each medication are shown along with the number of HAE-UNK patients treated by each expert. Each blue square represents the report by one expert. The size of the square is proportional to the number of HAE-UNK patients assessed, which is indicated next to it. The larger the square, the more patients showed the indicated response

For HAE-UNK (Fig. 4), the ODT efficacy of icatibant, used by 85 patients, was reported as high, moderate, and mild in 16.5%, 78.8%, and 4.7%, respectively. Plasma-derived C1INH, for ODT in 62 patients, was assessed as moderately effective in 16.1%, having mild efficacy in 74.2%, and not effective in 9.7%. Ecallantide was rated as highly efficacious for a single patient. As for LTP, subcutaneous plasma-derived C1INH was used by 25 patients, with efficacy reported as moderate and mild for 8% and 92%, respectively. Lanadelumab in 10 patients was assessed as moderately efficacious in seven and mildly efficacious in three. Berotralstat, in all 11 patients reported, was mildly effective. Attenuated androgens were used by six patients and were moderately effective, mildly effective, and non-effective in two each. The efficacy of antifibrinolytics, in 37 patients, was moderate in 13.5% and mild in 86.5%. Progestin treatment, in 15 patients, was assessed as mildly efficacious in 40% and non-effective in 60%.

### Further Real-World Studies and Clinical Trials in HAE-nC1INH are Needed

Information available on the treatment of patients with HAE-nC1INH remains limited highlighting the need for further investigations. These should include real-world studies making use of registries, patient-reported outcome measures, and multicenter approaches with high patient numbers. Clinical trials, while challenging in rare diseases, should be performed to assess the efficacy of existing and emerging treatment options.

The results of our HCT survey are in line with published observations, and they complement this information. Together, they suggest that plasma-derived C1INH and icatibant are both broadly used and effective ODTs in HAE-XII, HAE-PLG, and HAE-UNK. Ecallantide and recombinant C1INH, although perceived as effective, have only rarely been used by the clinicians surveyed. While it is counterintuitive that ODT with C1INH would be effective for diseases defined by normal C1INH levels, cleavage of C1INH to an inactive form during attacks has been documented [135, 136]. This finding has been interpreted as the explanation for the effectiveness of C1INH for ODT.

Information regarding STP outcomes in HAE-nC1INH is insufficient from the HCT survey and the literature to provide treatment recommendations. It is reasonable to consider the same approach as adopted for HAE-C1INH with treating physicians exercising clinical judgement. In all cases, it is recommended that ODT be made available for all patients.

Similar to HAE-C1INH, disease severity can be increased with estrogen exposure in HAE-nC1INH, in particular for HAE-XII. Given the negative disease impact, discontinuation of exogenous estrogen is recommended whenever possible [120]. In contrast to HAE-C1INH, progestin treatment has been effective in case series of HAE-FXII patients demonstrating a significant reduction in attacks [58, 120]. Similarly, all of the 29 HAE-XII patients in the HCT survey had moderate or better efficacy of progestin treatment. Taken together, LTP with progestin may be considered for HAE-FXII patients.

LTP with attenuated androgens in HAE-nC1INH, in the literature [120] and the HCT survey, is reported for only a limited number of patients with inconsistent efficacy. Given the known risk of side effects and inconsistent evidence of benefit, no recommendation can be made for their use in HAE-nC1INH.

LTP with antifibrinolytics appears to benefit patients with HAE-nC1INH, based on reports from the literature [52, 58, 120] as well as our HCT survey. Outcomes were particularly encouraging for HAE-PLG, where for all 12 of the patients, it was assessed as highly efficacious. A case series comparing antifibrinolytic and progestin therapy in HAE-PLG subjects found the former to be more effective [54, 58]. Results of the HCT survey provide additional evidence supporting the use of antifibrinolytics for LTP treatment in patients with HAE-PLG.

As of now, there are still very limited data on the use of lanadelumab for LTP in HAE-nC1INH. In the literature and in our HCT survey, the majority of the HAE-FXII patients who used it experienced high efficacy [115]. Patients with HAE-PLG, however, showed a much more mixed response to lanadelumab, with some patients experiencing moderate efficacy and others worsening. This may reflect that attacks in HAE-PLG relate to kinin generation directly from kininogen cleavage by plasmin, bypassing plasma kallikrein within the contact cascade [38]. HCT survey patients with HAE-UNK showed high efficacy of lanadelumab LTP in 70% and mild efficacy in 30%. Based on the currently available information, lanadelumab may be considered as an option for LTP in HAE-FXII and HAE-UNK, and it may help some patients with HAE-PLG.

The information on LTP with plasma-derived C1INH or berotralstat is limited; however, outcomes in HAE-nC1INH patients when treated were mostly favorable [120, 137].

## Conclusion and Forward-Looking Statement

The ability to unambiguously diagnose HAE-nC1INH currently relies on excluding other causes and identifying any underlying mutation; however, clinicians may not have access to gene sequencing. Many if not most patients with possible HAE-nC1INH do not have a demonstrated pathologic mutation. Even without an identified mutation, a presumptive diagnosis of HAE-nC1INH made by an expert physician is sufficient. Since these patients are at risk of serious morbidity and mortality, treatment needs to be available even for a presumptive diagnosis of HAE-nC1INH.

The inability to make definitive diagnoses severely limits our ability to conduct clinical trials to elucidate treatment options. Thus, we recommend that concerted efforts be made to validate accurate biomarkers that can improve diagnosis and treatment of these patients. Since HAE-nC1INH is a rare disease, national or international registries will be essential for learning more about the natural history and response to treatment [138]. The introduction of new techniques into genetic testing has increased the number of genes identified, and biorepositories create the means for analysis of recently identified genes in stored DNA samples of the patients [138, 139].

## Data Availability

No datasets were generated or analysed during the current study.

## Abbreviations

3-OST-6: HS-glucosamine 3-O-sulfotransferase 6
ACE: Angiotensin converting enzyme
AE-MC: Mast cell–mediated angioedema
AE-UNK: Angioedema unknown type
C1INH: C1 inhibitor
CPN: Carboxypeptidase N
DAB2IP: Disabled homolog 2 interacting protein
DPPIV: Dipeptidyl peptidase IV
*F12*: Coagulation factor XII gene
FXII: Coagulation factor XII
HAE: Hereditary angioedema
HAEA: US HAE Association
HAE-C1INH: HAE due to C1INH deficiency
HAEi: HAE International
HAE-nC1INH: HAE with normal C1INH
HAE-UNK: HAE of unknown cause
HCT: HAE-nC1INH Consensus Treatment
HK: High molecular–weight kininogen
HS: Heparan sulfate
INMA: Idiopathic non-mast cell-mediated angioedema
KKS: Kallikrein-kinin system
LK: Low molecular–weight kininogen
LTP: Long-term prophylaxis
NGS: Next-generation sequencing
NSAIDs: Non-steroidal anti-inflammatory drugs
ODT: On-demand treatment
STP: Short-term prophylaxis
TIE2: Tunica internal endothelial cell kinase 2
VEGF: Vascular endothelial growth factor
VEGFR2: VEGFR receptor 2
